# SOD1-related inherited peripheral neuropathies in a Japanese cohort: genetic variants and clinical insights

**DOI:** 10.1007/s00415-025-12925-4

**Published:** 2025-02-11

**Authors:** Masahiro Ando, Yujiro Higuchi, Jun-Hui Yuan, Akiko Yoshimura, Chikashi Yano, Takahiro Hobara, Fumikazu Kojima, Yu Hiramatsu, Satoshi Nozuma, Tomonori Nakamura, Yusuke Sakiyama, Akihiro Hashiguchi, Yuji Okamoto, Takeshi Matsushige, Jun Mitsui, Shoji Tsuji, Hiroshi Takashima

**Affiliations:** 1 Department of Neurology and Geriatrics, Kagoshima University Graduate School of Medical and Dental Sciences, 8-35-1 Sakuragaoka, Kagoshima City, Kagoshima 890-8520 Japan; 2 Department of Physical Therapy, Kagoshima University of School of Health Sciences, Kagoshima, Japan; 3 Department of Pediatrics, Yamaguchi University Graduate School of Medicine, Yamaguchi, Japan; 4 Department of Precision Medicine Neurology, Graduate School of Medicine, The University of Tokyo, Tokyo, Japan; 5 Department of Neurology, The University of Tokyo Hospital, Tokyo, Japan; 6 Institute of Medical Genomics, International University of Health and Welfare, Chiba, Japan

**Keywords:** Inherited peripheral neuropathy, SOD1, Amyotrophic lateral sclerosis, Electrophysiology

## Abstract

**Background:**

Inherited peripheral neuropathies (IPNs) encompass a wide range of disorders affecting the peripheral nervous system, often with complex genetic causes and frequent underdiagnosis. The variants in the superoxide dismutase 1 (*SOD1*) gene, primarily linked to amyotrophic lateral sclerosis (ALS), have also been associated with peripheral neuropathy. The recent approval of Tofersen, targeting *SOD1*-related ALS, highlights the importance of precise genetic diagnosis. This study explores the clinical and genetic profiles of *SOD1*-related IPNs (*SOD1*-IPN) in a nationwide Japanese IPN cohort.

**Methods:**

Clinical and genetic data were assessed from 1483 Japanese patients with IPN, with a focus on those harboring *SOD1* pathogenic variants. The clinical evaluations included age of onset, gender, muscle weakness patterns, sensory disturbances, reflex responses, and electrophysiological findings.

**Results:**

Seventeen patients with *SOD1* pathogenic variants were identified, reinforcing *SOD1*’s role in IPN. The average onset age was 47, with a slight male predominance. Distal muscle weakness was noted in 9 of 13 patients, and asymmetric muscle weakness and atrophy in 10 of 14 cases. Mild sensory disturbances were observed in eight patients, with some showing hyperreflexia and abnormal reflexes. Electrophysiology predominantly indicated a length-dependent, motor-dominant axonal neuropathy.

**Conclusion:**

This study reveals the clinical variability and likely underdiagnosis of *SOD1*-IPN, supporting the integration of *SOD1* screening in IPN genetic testing, especially for patients with asymmetric, length-dependent axonal neuropathy evident in clinical and electrophysiological assessments.

**Supplementary Information:**

The online version contains supplementary material available at 10.1007/s00415-025-12925-4.

## Introduction

Inherited peripheral neuropathies (IPNs) are a diverse group of neurological conditions that primarily impact the peripheral nervous system. This group includes several distinct clinical types: Charcot–Marie–Tooth disease (CMT), also known as hereditary motor and sensory neuropathy (HMSN), hereditary motor neuropathy (HMN), and hereditary sensory (and autonomic) neuropathy (HSN/HSAN). CMT is the most prevalent, marked by progressive motor and sensory impairment, foot deformities, and diminished tendon reflexes. In contrast, HMN primarily affects motor function, with minimal sensory involvement. The complex genetic background of IPNs, involving over 140 genes, contributes to a significant proportion of genetically undiagnosed IPN cases [1].

Superoxide dismutase 1 (*SOD1*) is a rare but noteworthy gene linked to IPN. Although *SOD1* is primarily recognized for its role in amyotrophic lateral sclerosis (ALS), a progressive motor neuron disease, pathogenic variants in this gene have also been associated with certain IPN forms, particularly CMT and HMN [2–5]. *SOD1* pathogenic variants were the first genetic cause identified for familial ALS, accounting for about 15–25% of familial ALS cases and 1–2% of ALS cases overall [6, 7]. The *SOD1* protein is a widely expressed cytosolic enzyme that forms homodimers and catalyzes the conversion of superoxide radicals into hydrogen peroxide and oxygen. Initially, *SOD1* pathogenic variants in ALS were thought to result from a loss of enzymatic function and increased reactive oxygen species production. However, later research indicates that *SOD1*-related ALS is mainly driven by a toxic gain-of-function mechanism [8–10].

Therapeutic strategies are being developed for *SOD1*-related ALS. Tofersen, an antisense oligonucleotide, reduces *SOD1* expression by promoting the ribonuclease (RNase)-mediated degradation of *SOD1* mRNA (messenger RNA), showing significant effects on ALS biomarkers [11, 12]. Consequently, early diagnosis of *SOD1*-related IPNs has gained importance. This study aims to investigate the clinical and genetic characteristics of Japanese patients with *SOD1* pathogenic variants within an IPN cohort.

## Materials and methods

### Enrollment criteria

We performed detailed clinical and genetic analyses on Japanese patients participating in a nationwide genetic study on IPNs/CMT. For all patients with the demyelinating type, *PMP22* (peripheral myelin protein 22) duplication/deletion was ruled out using fluorescence in situ hybridization or multiple ligation-dependent probe amplification. This Institutional Review Board of Kagoshima University approved this study (Application ID: 490) and informed consent was obtained from all patients and their family members before study participation. The clinical and electrophysiological data were collected by reviewing patient records and assessments from the medical facilities involved.

### Genetic analysis

**Fig. 1:**
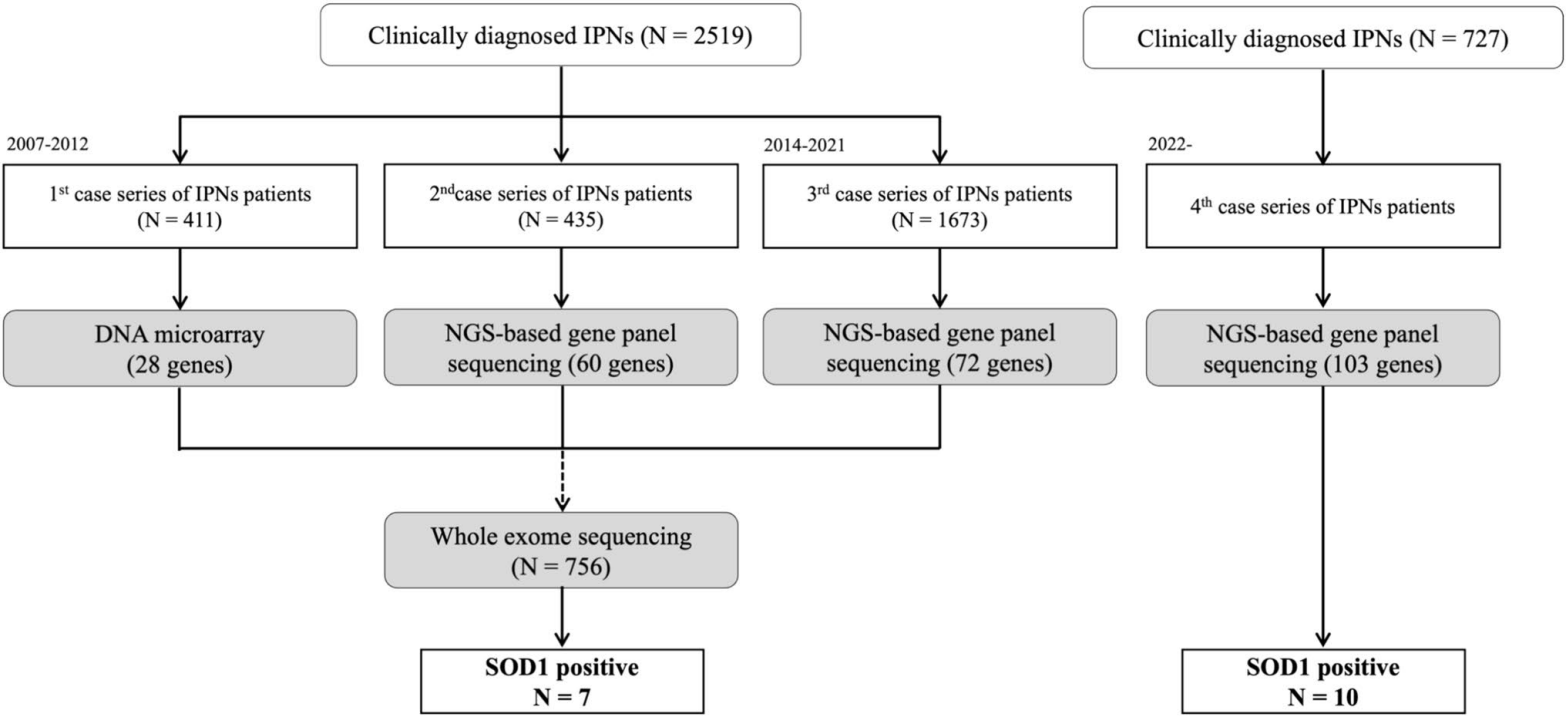
Flowchart of *SOD1* analyses in the Japanese IPN/CMT case series. Comprehensive genetic testing was conducted on 2519 Japanese patients with a clinical diagnosis of IPNs. The analysis included DNA microarrays, next-generation sequencing-based gene panel sequencing, and whole-exome sequencing (WES). An additional gene panel analysis covering 103 IPNs/CMT-related genes, including SOD1, was performed in 727 patients from 2022 onward. SOD1 mutations were identified in 17 patients

Between 2007 and 2021, 2519 Japanese patients clinically diagnosed with IPNs/CMT were screened using in-house gene panel sequencing for IPNs/CMT-related genes via DNA microarrays (Affymetrix, Santa Clara, CA, USA), Illumina MiSeq (Illumina, San Diego, CA, USA), or ion proton (Thermo Fisher Scientific, Waltham, MA, USA). Importantly, *SOD1* was not included in any of these three panels. Whole-exome sequencing was performed on a subset of 756 patients who tested negative in the initial panel screenings. Since 2022, an updated panel with 103 genes relevant to IPNs/CMT, including *SOD1*, has been used, allowing the screening of an additional 727 Japanese patients. The study’s methodology is outlined in Fig. 1.

The sequence data were aligned to the human genome reference (NCBI37/hg19) and variant calling was conducted using Burrows–Wheeler Aligner and SAMtools. Variants were annotated with CLC Genomics Workbench software (Qiagen, Hilden, Germany) and an in-house script. The pathogenic variants previously reported were verified by consulting the Human Gene Mutation Database Professional. Novel variants were compared against global population databases (Genome Aggregation Database [gnomAD]), the Japanese control database (jMorp), and our internal control database. The variants were assessed using four in silico prediction tools: FATHMM, CADD, REVEL, and BayesDel. All potential disease-causing variants were validated by Sanger sequencing and interpreted following the American College of Medical Genetics and Genomics (ACMG) standards and guidelines. These methods have been previously outlined [13]. The numbering of SOD1 variants in this study adheres to the recommendations of the Human Genome Variation Society (HGVS) for variant nomenclature.

## Results

### Genetic findings

In our cohort, 17 patients were identified with previously reported *SOD1* variants. These variants included p.Leu9Val (one case), p.Gly38Arg (one case), p.His47Arg (four cases), p.Gly94Ser (two cases), p.Ile114Thr (one case), p.Glu122Gly (one case), p.Asp126Asn (one case), p.Leu127Ser (five cases), and p.Leu145Ser (one case). Additionally, a previously identified co-occurring *MFN2* pathogenic variants, p.Arg94Trp, was found in Patient 2 alongside the *SOD1* variants, p.Gly38Arg.

In addition, to the known variants, two novel variants were identified: p.Val15Ala and p.Arg144Cys. These variants were evaluated following the ACMG guidelines. Previous reports have described different amino acid substitutions at the same residue (PM5). In silico predictions indicated that these variants were likely damaging. However, they were found in both gnomAD and jMorp with allele frequencies higher than expected for *SOD1*-IPN (BS1). Based on these findings, the variants were classified as having uncertain significance according to the ACMG guidelines. More details on these variants are available in the supplementary table. These novel variants were excluded from the clinical and electrophysiological analyses.

### Clinical findings

The clinical features of the 17 patients were summarized, including the case with digenic pathogenic variants in the *MFN2* and *SOD1* genes (Table 1). The mean age of onset, based on the 16 patients excluding the digenic case, was 47 ± 15 years, with nine males and seven females. A family history of neuropathy was reported in 10 of the 16 cases. The initial symptoms, primarily muscle weakness or related manifestations, were observed in 13 of 15 patients. One patient presented with myalgia, while another had numbness. Additionally, two patients experienced both muscle weakness and sensory disturbances at the onset.

**Table 1:** Clinical summary of 17 patients with SOD1-IPN

Patients number	1	2	3	4	5	6	7	8	9	10	11	12	13	14	15	16	17
Variants	p.Leu9Val	p.Gly38Arg + MFN2 mut	p.His47Arg	p.His47Arg	p.His47Arg	p.His47Arg	p.Gly94Ser	p.Gly94Ser	p.Ile114Thr	p.Glu122Gly	p.Asp126Asn	p.Leu127Ser	p.Leu127Ser	p.Leu127Ser	p.Leu127Ser	p.Leu127Ser	p.Leu145Ser
Onset	43	< 10	46	40	51	36	29	38	75	68	65	33	35	30	43	70	56
Exam age	53	< 10	50	43	52	42	32	39	82	76	67	36	39	70	56	77	61
Gender	F	M	M	M	M	M	F	M	F	F	M	F	M	M	M	F	F
Family history	+	+	–	+	+	+	–	–	+	+	+	+	+	+	–	–	–
Initial symptom	Numbness	Impaired running performance	Muscle weakness (upper limb)	Muscle weakness (lower limb)	Muscle weakness (lower limb)	Cramp, difficulty in ankle plantarflexion and left calf atrophy	Muscle weakness (lower limb)	Leg weakness, myalgia	Muscle weakness (upper limb)	Fine motor impairment:	Cramp, pain, numbness muscle weakness	Gait disturbance	Myalgia	n.a	Muscle weakness (lower limb)	Muscle weakness (lower limb), sensory disturbance	Muscle weakness (lower limb)
Upper limb weakness	–	–	+	+	–	–	+	–	+	+	+	+	–	–	–	+	+
Upper limb proximal MMT	n.a	n.a	4	5	5	n.a	5	5	4	5	4	4	5	n.a	n.a	5	3
Upper limb distal MMT	n.a	n.a	4	4	5	n.a	3	5	2	2	3	5	5	n.a	n.a	3	3
Lower limb weakness	+	+	+	+	+	+	+	+	+	+	+	+	+	+	+	+	+
Lower limb proximal MMT	n.a	n.a	4	4	5	n.a	5	4	4	5	3	4	4	n.a	4	4	2
Lower limb distal MMT	n.a	4	2	2	4	n.a	3	4	2	4	2	4	4	n.a	3	3	2
Muscle atrophy	n.a	n.a	+	+	–	+	+	+	+	+	+	−	+	n.a	+	+	+
Pes caves	n.a	+	n.a	n.a	n.a	+	n.a	n.a	n.a	+	n.a	n.a	+	n.a	+	n.a	n.a
Laterality	n.a	n.a	L	R	R	L	R	L	R	L	–	R	–	n.a	–	–	R
Muscle cramp/ fasciculation	n.a	n.a	−	+	n.a	+	+	–	+	+	+	–	+	n.a	–	–	–
Sensory disturbance	+	n.a	+	–	–	–	–	–	+	–	–	+	+	+	–	+	+
Upper limb DTR	Hyper	n.a	Absent	Normal	Normal	n.a	Normal	Normal	Absent	Hyper	Normal	Hyper	Hyper	Decrease	Normal	Normal	Normal
Lower limb DTR	Absent	n.a	Absent	Absent	Normal	Absent	Decrease	Absent	Absent	Absent	Absent	Hyper	Absent	Decrease	Normal	Absent	Absent
Pathological reflex	+	n.a	–	+	–	–	+	+	+	–	–	+	–	–	–	–	–
Bulbar symptom	–	n.a	–	–	–	–	–	–	–	–	–	–	–	n.a	n.a	–	–
Respiratory failure	n.a	–	n.a	–	–	n.a	n.a	–	–	n.a	–	n.a	–	n.a	n.a	n.a	n.a
Autonomic dysfunction	–	n.a	–	–	–	n.a	n.a	–	–	n.a	–	–	–	n.a	n.a	–	–
Serum-CK	50	n.a	700–1400	1115	450	710	372	n.a	58	n.a	300	n.a	213	n.a	n.a	n.a	n.a

MMT: manual muscle test, DTR: deep tendon reflex, n.a: not available, R: right-side predominance, L: left-side predominance

Upper limb muscle weakness was noted in 9 out of the 16 cases, while all patients had lower limb muscle weakness. Muscle atrophy was observed in 12 of the 14 patients and 7 of the 13 patients experienced fasciculations or muscle cramps. Additionally, pes cavus was seen in five patients. Notably, significant asymmetry in muscle weakness and atrophy was common, with 10 of the 14 cases showing asymmetric involvement. Right-sided predominance was noted in six cases and left-sided predominance in four cases.

Regarding tendon reflexes, hyperreflexia in the upper limbs was observed in four patients, normal reflexes in eight patients, and decreased reflexes in three patients. In the lower limbs, 13 of the 16 patients showed decreased tendon reflexes. The pathological reflexes were found in six cases. Sensory disturbances, generally mild, were seen in 8 out of 16 patients. Notably, none of the patients exhibited bulbar symptoms, respiratory failure, or autonomic dysfunction. Serum creatine kinase levels were elevated with a mean value of 441 ± 348 U/L.

### Electrophysiological findings

The median motor nerve conduction velocity (MNCV) averaged 55.2 m/s, with all cases classified as axonal neuropathy. The median compound muscle action potential (CMAP) was reduced in three patients. The tibial MNCV averaged 43.7 m/s, within the normal range, while the mean tibial CMAP was reduced to 2 mV, with normal values in three cases and decreased values in 11 cases. The sural sensory nerve action potential (SNAP) was generally preserved with a mean of 12.6 μV, though reduced in four cases and unrecordable in one.

Needle electromyography (EMG) was performed in 10 patients, all of whom showed chronic neurogenic changes primarily in the lower limb muscles. Three patients displayed active denervation signs, including fibrillation potentials, positive sharp waves, and fasciculation potentials. Detailed NCS/nEMG information is summarized in Table 2 and 3.

**Table 2:** Electrophysiological findings in 17 patients with SOD1-IPN

Patients number		1	2	3	4	5	6	7	8	9	10	11	12	13	14	15	16	17
Variants		p.Leu9Val	p.Gly38Arg + MFN2 mut	p.His47Arg	p.His47Arg	p.His47Arg	p.His47Arg	p.Gly94Ser	p.Gly94Ser	p.Ile114Thr	p.Glu122Gly	p.Asp126Asn	p.Leu127Ser	p.Leu127Ser	p.Leu127Ser	p.Leu127Ser	p.Leu127Ser	p.Leu145Ser
Median	MNCV	59	54	NE	52.8	n.a	63.9	53.9	55	n.a	51.1	47	54	58	56	63.4	49.9	54
	CMAP	19	6.3	NE	3.6	n.a	10.5	0.8	10.1	n.a	2.1	6.1	5.6	5.4	9.5	5.7	3.8	6
Ulnar	MNCV	60	52.1	45.6	50	59.6	63.2	55.8	68.4	n.a	60.3	56	68	60	56	n.a	n.a	63
	CMAP	7.6	4.4	2.6	5.1	4.2	11.9	5.3	20.2	n.a	3.6	8.2	9.3	8.2	7.6	n.a	n.a	10.1
Tibial	MNCV	48	33.9	34.9	47.2	49.4	50.3	NE	44.3	n.a	41.2	42	51	43.8	n.a	40	37	39
	CMAP	4.4	1	0.1	0.97	10	0.63	NE	0.64	n.a	3.5	0.07	5	2.2	n.a	0.2	0.5	0.06
Median	SCV	49	42.9	44.2	59.1	57.3	60.2	61.1	63.1	n.a	51.6	49	52	57.5	n.a	52.5	59.4	45.5
	SNAP	28	7.2	14	27.8	4.3	35.7	31.1	30.7	n.a	19.8	6.6	42	25	n.a	48	n.a	9.2
Ulnar	SCV	47	n.a	48.7	52.4	n.a	59.2	52.4	63.4	n.a	53.4	42	56	n.a	n.a	n.a	n.a	56.2
	SNAP	77	n.a	18	12.7	n.a	21.5	62.5	18.2	n.a	10.6	8.8	56	n.a	n.a	n.a	n.a	3.1
Sural	SCV	NE	NE	48.1	44.6	52	50	49	40.1	n.a	40.5	44	55	45.4	n.a	44	47.5	48.6
	SNAP	NE	NE	6.6	20.7	3.5	18	31.2	15.6	n.a	14.9	4.4	11	31	na	4.8	n.a	2.4

MNCV: Motor nerve conduction velocity; CMAP: Compound motor action potential; SCV: Sensory nerve conduction velocity; SNAP: Sensory nerve action potential; n.a.: Not available; NE: Not evoked; Normal range: Median CMAP > 3.1 mV; Median MNCV > 49.6 m/s; Median SNAP > 7.0 μV; Median SCV > 47.2 m/s; Ulnar CMAP > 6.0 mV; Ulnar MNCV > 50.1 m/s; Ulnar SNAP > 6.9 μV; Ulnar SCV > 46.9 m/s; Tibial CMAP > 4.4 mV; Tibial MNCV > 41.7 m/s; Sural SNAP > 5.0 μV; Sural SCV > 40.8 m/s

**Table 3:** Needle EMG Findings in Patients with SOD1 variants

Patients number	Variants	Needle EMG Observations
3	p.His47Arg	Active and chronic neurogenic change (Upper and lower limb, proximal and distal)
4	p.His47Arg	Chronic neurogenic change (FDI, TA), Normal (BB)
5	p.His47Arg	Chronic neurogenic change (Lower limb)
6	p.His47Arg	Chronic neurogenic change (Lower limb)
7	p.Gly94Ser	Active (fibs/PSW) and chronic neurogenic change (FDI, VM, TA), Normal (BB, SCM)
8	p.Gly94Ser	Active (fibs/PSW/FSP) and chronic neurogenic change (Lower limb)
9	p.Ile114Thr	Chronic neurogenic change (TB, VM, TA)
12	p.Leu127Ser	Chronic neurogenic change (TA), Normal (IP, FDI, BB)
15	p.Leu127Ser	Chronic neurogenic change (lower limb proximal/distal), Normal (upper limb proximal/distal)
16	p.Leu127Ser	Chronic neurogenic change (TA, GC, VM)

FDI: first dorsal interosseous, TA: tibialis anterior, BB: biceps brachii, VM: vastus medialis, TB: triceps brachii, SCM: sternocleidomastoid, IP: iliopsoas, GC: gastrocnemius, fibs: fibrillation potentials, PSW: positive sharp waves, FSP: fasciculation potentials

## Discussion

This study provides an in-depth clinical and genetic analysis of *SOD1*-IPN in a Japanese cohort, contributing to one of the largest genetic diagnostic studies on Japanese IPN/CMT patients. The study successfully identified 17 cases through genetic testing, offering valuable insights into the phenotypic, genotypic, electrophysiological, and clinical profiles associated with these pathogenic variants.

The *SOD1* gene, first identified in 1993 as a cause of familial ALS [6], encodes a 154-amino acid protein that acts as a catalytic enzyme to convert superoxide radicals into oxygen and hydrogen peroxide, crucial for cellular defense against oxidative stress. A total of 208 pathogenic variants in the *SOD1* gene have been reported to date (ALSoD [https://alsod.ac.uk], accessed 2024/10/29).

While the involvement of *SOD1* pathogenic variants in ALS is well established, their association with IPNs is less recognized. The first mention of *SOD1*-related neuropathy appeared in 2003, when two familial ALS cases were found to have concurrent sensory neuropathy [14]. In 2012, a case of HMN linked to a *SOD1* pathogenic variants was reported, followed by another in 2022 describing HMSN associated with a *SOD1* pathogenic variants [3, 5]. These reports, although rare, connected *SOD1* to the peripheral nervous system. However, in our IPN cohort, we identified *SOD1* pathogenic variants in 17 cases, suggesting that *SOD1* pathogenic variants may be underrecognized as a cause of IPN. This underreporting could be due to *SOD1* not being routinely included as a target in genetic testing for IPN, leading to its exclusion from many diagnostic panels.

Therapeutic strategies for *SOD1*-related conditions are progressing, with Tofersen being a notable example. This antisense oligonucleotide works by reducing the expression of the mutant *SOD1* protein through RNase-mediated degradation of its mRNA. The clinical trials have shown Tofersen’s significant impact, especially in ALS, by lowering biomarkers associated with disease progression [11, 12]. Due to its therapeutic potential, it is important to include *SOD1* screening in genetic tests for patients with IPN.

The average age of onset in our cohort was 47 years, which is similar to the mean age of onset for *SOD1*-ALS, reported as 48.9 years [15]. The male-to-female ratio in our cohort was 9:7, reflecting a slight male predominance, consistent with findings in *SOD1*-ALS [15]. ALS generally has a higher incidence in males, and studies on *SOD1* (G93A) rat models have shown increased cytotoxicity in male neural progenitor cells due to *SOD1* overexpression, which may contribute to this trend [16]. In our cohort of 16 SOD1-IPN cases, the average duration from onset to examination was 7 years, with the longest duration reaching 40 years. This slow progression is notably different from the typical course of ALS, which is characterized by a more rapid progression. All patients in our cohort exhibited muscle weakness, with lower limb involvement in all cases and more than half also showing weakness in the upper limbs. Of the 13 patients with manual muscle testing data, 9 showed distal-predominant weakness, in line with typical IPN/CMT presentations. A notable characteristic in our cohort was significant asymmetry in muscle weakness and atrophy, observed in 10 out of 14 cases. While IPN is typically symmetric, significant asymmetry has been observed in conditions like CMT with *MORC2* pathogenic variants [17], which may help differentiate *SOD1*-IPN in the differential diagnosis.

Sensory deficits are not commonly associated with ALS, but *SOD1* pathogenic variants have been linked to sensory involvement. In addition, the patients with both motor and sensory symptoms, diagnosed with HMSN/CMT, have been found to carry *SOD1* pathogenic variants [5, 14]. In this study, mild sensory disturbances were observed in 8 out of 16 patients, with 1 patient presenting with numbness and another reporting both muscle weakness and sensory issues at disease onset. While sural SNAPs were generally preserved, abnormalities were noted in a subset, including one patient with an unrecordable sural SNAP and reductions in four others. These findings indicate that *SOD1*-related neuropathies may present with more complex neurodegenerative features than previously thought, blurring the lines between motor-dominant disorders like ALS and sensorimotor neuropathies like CMT.

The presence of hyperreflexia (in four cases) and pathological reflexes (in six cases) adds complexity to the diagnosis, as these signs are typically associated with upper motor neuron involvement, a hallmark of ALS. Notably, such reflex abnormalities have also been observed in subtypes of HMSN, particularly in HMSN-V, which involves both motor and sensory deficits. This overlap suggests that in some patients, *SOD1*-IPN may present similarly to the HMSN-V phenotype. Importantly, in our cohort, we did not identify bulbar symptoms or respiratory failure, which are commonly observed in ALS cases.

Electrophysiological evaluations revealed an axonal neuropathy pattern, with preserved CMAPs in the upper limbs and reduced CMAPs in the lower limbs, indicating a length-dependent neuropathy. This pattern, where CMAPs remain relatively intact in the upper limbs, differs from typical ALS, where motor neuron degeneration typically causes a generalized reduction in CMAPs across all limb regions without a clear length-dependent distribution. Conversely, the selective involvement of the lower limbs aligns more with patterns seen in CMT, especially in axonal forms like CMT2. This distinct distribution of CMAP abnormalities may provide a useful diagnostic feature to differentiate *SOD1*-ALS from *SOD1*-IPN.

Active denervation signs, typically associated with ALS, can also be found in CMT, especially in advanced stages, although they are generally less severe and widespread compared to ALS. In this study, EMG revealed chronic neurogenic changes primarily in the lower limbs, with signs of active denervation, including fibrillation potentials, positive sharp waves, and fasciculation potentials, in 3 out of 10 cases. Interestingly, some upper limb muscles showed no abnormal findings. This distribution supports the diagnosis of length-dependent peripheral neuropathy and is consistent with the clinical presentation of *SOD1*-IPN. These EMG results emphasize the value of comprehensive electrophysiological evaluation in *SOD1* pathogenic variants patients to help distinguish *SOD1*-IPN from ALS.

In our cohort, only one case (Patient 3) exhibited active denervation in multiple regions of both the upper and lower limbs. However, this patient did not display upper motor neuron signs but presented with sensory impairments and distal muscle weakness, a pattern characteristic of CMT. In the remaining cases, signs of active denervation were either absent or confined to specific regions. The absence of bulbar symptoms, respiratory failure, and the presence of sensory impairments, along with the electrophysiological findings, collectively distinguishes SOD1-IPN from ALS.

A limitation of this study is the variability in the clinical data due to its collection from multiple medical institutions, which led to inconsistencies in the availability and completeness of certain clinical details. For instance, detailed information regarding distal muscle weakness was not uniformly available for all patients, as this study relied on medical records and assessments from various sources.

Additionally, differences in the electrophysiological procedures used across institutions may have introduced operational variability, potentially influencing the results and clinical diagnoses. Furthermore, the retrospective nature of this study limited the availability of longitudinal follow-up data, such as survival outcomes and disease progression patterns. This constraint hinders a comprehensive characterization of the long-term clinical course of the identified patients.

In conclusion, our study identified 17 cases of *SOD1*-IPN within the cohort, highlighting the significant role of *SOD1* pathogenic variants as a cause of IPN. This emphasizes the importance of including *SOD1* analysis in genetic testing for IPN, particularly in patients showing asymmetric, length-dependent axonal neuropathy, as noted in both clinical and electrophysiological findings. Furthermore, our results highlight the phenotypic variability of *SOD1*-IPN, which may present in various forms, including HMN, CMT/HMSN, or even the more complex HMSN-V phenotype. Accurate genetic diagnosis is essential for guiding treatment, especially with emerging therapies that may offer new options for *SOD1*-related conditions.

## Supplementary Information

Below is the link to the electronic supplementary material.

Supplementary file 1: Table 1 Genetic findings of SOD1 p.Val15Ala and p.Arg144Cys

## Data Availability

Datasets are not readily available due to ethical and privacy restrictions. Requests should be directed to the corresponding author.
